# Hypoxia Confers Tumor with a Higher Immune Infiltration but Lower Mutation Burden in Gastrointestinal Cancer

**DOI:** 10.1155/2022/4965167

**Published:** 2022-09-12

**Authors:** Junjie Hu, Wangxiong Hu, Yanmei Yang

**Affiliations:** ^1^Key Laboratory of Reproductive and Genetics, Ministry of Education, Women's Hospital, Zhejiang University School of Medicine, Hangzhou, Zhejiang 310006, China; ^2^Cancer Institute, Key Laboratory of Cancer Prevention and Intervention, China National Ministry of Education, The Second Affiliated Hospital, Zhejiang University, School of Medicine, Hangzhou, Zhejiang 310009, China

## Abstract

**Background:**

Hypoxia is one of the driving forces of cancer progression, recurrence, and metastasis. However, the association between the tumor hypoxic tumor microenvironment and the tumor mutation burden (TMB) is poorly understood in gastrointestinal cancer.

**Methods:**

Approximately 2,000 samples from colorectal cancer (CRC) and stomach adenocarcinoma (STAD) patients were obtained from the gene expression omnibus database and the cancer genome Atlas databases and were clustered and subtyped by nonnegative matrix factorization. Significant differentially expressed genes that were possibly related to survival differences between the hypoxic and normoxic groups were subjected to multivariate Cox regression.

**Results:**

Gastrointestinal cancer patients with CRC and STAD were further divided into two subgroups, namely, the hypoxia group and the normoxia group, and hypoxia was correlated with unfavorable outcomes. Notably, hypoxic tumors had lower TMB but significantly higher levels of immune and stromal infiltration. A signature of *HEYL* and *NRP1* selected by LASSO classified gastrointestinal cancer patients into either a low or high-risk group, allowing for the combination of TMB status with markers of hypoxia in future clinical applications.

**Conclusions:**

Hypoxia is an independent prognostic factor and a strong immune infiltration indicator in gastrointestinal tumors of different organs, especially for cancers with low TMB.

## 1. Introduction

The hypermutated (>10 mutations per Mb) or microsatellite instability-high (MSI-H, relatively prevalent in gastrointestinal cancer) tumors generally have a very high rate of nonsilent mutations (nonsynonymous, indel frameshift, nonsense, and dys-splicing), leading to an increased number of neoepitopes and cytotoxic T lymphocytes (CTLs), thus termed ‘hot' tumors [1]. This is one of the important reasons why a high tumor mutational burden (TMB) in patients predict a good clinical response to an immune checkpoint blockade (ICB) and prolonged overall survival (OS) [2]. Nevertheless, after ICB treatment, less than half of MSI-H patients achieved a clinical complete response (CR) or partial response (PR) despite a high level of immune cell infiltration within the tumor microenvironment (TME). In contrast, some patients with microsatellite stable (MSS) or nonhypermuted tumors also gain survival benefits or symptom alleviation from ICB treatment, suggesting that the relationship between TMB and immune status is more complicated than our expectations. Thus, an in-depth inspection of the association between TMB and the immune score is urgently required.

Hypoxia, as an important external factor within TME, has a close association with immunosuppression [3]. The antitumor immune response can be suppressed by hypoxia because nonspecific CD4+ T cells differentiate into regulatory T-cells (CD4 + CD25^High^FOXP3+) or *T* helper cells (Тh17) in the presence of hypoxia-inducible factor 1 (HIF-1) [4], an important transcription factor induced by hypoxia. Regulatory T cells repress effector T cell function via producing extracellular adenosine under hypoxic conditions. Thus, hypoxia resembles TMB and plays a pivotal role in orchestrating immune status but needs further elucidation.

Here, 1,043 RNA-seq samples from patients with colorectal cancer (CRC) and stomach adenocarcinoma (STAD) obtained from the cancer genome Atlas (TCGA) were clustered and subtyped by nonnegative matrix factorization (NMF) based on a custom hypoxia signature. We found that gastrointestinal cancer could be mainly categorized into two subgroups across tissue specificity, namely, the hypoxia group and the normoxia group. In addition, hypoxia tended to induce tumors with low TMB, but these tumors had significantly higher immune and stromal cell infiltration than normoxic tumors, including both the normoxic hypermutated and nonhypermutated tumor subtypes. Further, an inspection revealed that terminally exhausted CD8 (exhCD8) T cells were enriched under hypoxic conditions. As such, hypoxia may play an important role in reshuffling the TME and reconciling the overall high immune score and treatment resistance. Meanwhile, hypoxia was correlated with poor patient outcomes, and we established a two-gene signature (*HEYL* and *NRP1*) by least absolute shrinkage and selection operator (LASSO) that could predict the prognosis of patients with hypoxic gastrointestinal cancer. Based on our findings, hypoxia is a promising candidate marker for determining immunotherapy response either alone or in combination with other therapies, such as radiotherapy and chemotherapy, especially for patients with low TMB.

## 2. Materials and Methods

### 2.1. Definition 1

of hypermutated gastrointestinal cancer

The somatic mutational profiles of CRC and STAD called by the Mutect method were retrieved from the TCGA database (06/02/2018). RNA, silent, intronic, 5′ untranslated regions (UTR), 3′UTR, and flanking sequence mutations were discarded. Hypermutated tumors were designated as those with >10 mutations per Mb. As described by Thorsson et al. [5], tumor neoantigens were predicted. MSI-H status was defined as described earlier[6].

### 2.2. Gene Expression Data Processing and Normalization

The level 3 mRNA RNASeqV2 datasets for the CRC and STAD patient samples were downloaded from the TCGA website (09/12/2017). Genes with expression levels <1 (RSEM-normalized) in more than 50% of the samples were removed. Raw CEL files of the GSE39582 and GSE62254 (Affymetrix HG U133 Plus 2.0 arrays) datasets were downloaded from the GEO database. The MAS5 algorithm was used to determine the gene expression levels as previously described [7]. Known batch effects were corrected using the *ComBat* function of the Bioconductor *sva* package [8]. Infiltration of stromal and immune scores and cells in malignant tumor tissues was performed by the *estimate* package in the R and xCell methods, respectively [9]. Single-sample GSEA (ssGSEA) was applied to evaluate the terminally exhausted CD8 T cell enrichment scores using the GSVA package [10]. Principal component analysis (PCA) was performed by the *prcomp* function. Analysis of differentially expressed genes (DEGs) was performed using the *DEGSeq* package for R/Bioconductor [11]. Significant DEGs were selected according to a false discovery rate (FDR)-adjusted *P* value < 0.05 and fold change >2 conditions. All heatmaps were generated using the *pheatmap* package in R (64-bit, version 3.0.2).

### 2.3. Subtyping of Gastrointestinal Cancer According to Hypoxic Signature

A custom-built356-gene hypoxia signature including ADM, ANGPTL4, CA9, and VEGFA was curated as previously described [12]. Subsequent NMF clustering of gastrointestinal samples was based on the expression of 356 hypoxia-related genes with equal weight. NMF was performed by the *NMF* package in R [13].

### 2.4. Survival Analysis

Survival differences between the hypoxic and normoxic groups were tested by the Kaplan–Meier method and analyzed as previously described [6].

### 2.5. Cytotoxic Experiment of Effector CD8 T-Cell under Hypoxic Conditions

The HCT116 cell line was purchased from ATCC and cultured with RPMI 1640 medium (Gibco) containing 10% fetal bovine serum (FBS, BI Industry). CD8^+^T-cells were isolated from peripheral blood mononuclear cells (PBMCs) and stimulated by IL2 (SinoBiological, Catalog Number: GMP-11848-HNAE-B) and OKT3 (BD Pharmingen, Catalog Number: 566685) as described in our previous work [14]. Effector T-cells were cocultured with HCT116 cells for 48 hours in a 96-well plate. The cells were incubated at 37°C with 5% CO_2_ or hypoxic conditions (1% O_2_, BioSpherix). Specific lactic dehydrogenase (LDH) released from tumor cells in cell-free supernatant was detected using a cytotoxicity LDH detection kit (Genmed), following the manufacturer's instructions. The amount of LDH released was used to assess the lysis of target cells, which can be translated into the lethal effect of effector cells. Percent cytotoxicity was calculated according to OD values using the following formula:

Cytotoxicity % = (Experimental-Effectorspontaneous-Target spontaneous)/(Target maximum-Target spontaneous)×100%.

## 3. Results

### 3.1. The CRC and STAD Gastrointestinal Cancer Samples Mainly Clustered into Two Hypoxic Subtypes

**Figure 1:**
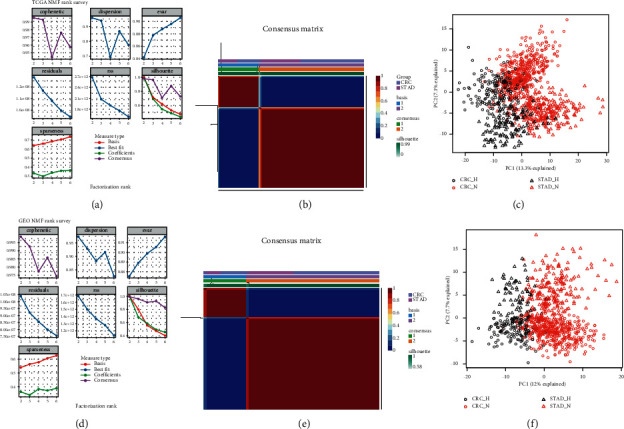
Gastrointestinal cancer clustered into two subtypes based on hypoxic signature. (a) Rank survey of the parameter (r) in TCGA samples by NMF. (b) Clustering of 1,043 gastrointestinal cancers in the TCGA by NMF. (c) PCA plot of TCGA samples according to the subtypes identified by NMF. (d) Rank survey of the parameter (r) in GEO samples by NMF. (e) Clustering of 885 gastrointestinal cancers in the GEO by NMF. (f) PCA plot of GSE39582 and GSE62254 samples.

Based on the tailored 356-gene hypoxic signature, the NMF algorithm was used to determine whether any clusters were present in gastrointestinal cancer (CRC and STAD) as described earlier[15]. In brief, to choose the best factorization rank *r*, which is a critical parameter in NMF, values of 2–6 were successively calculated in ascending order. Then, according to the first value of *r* at which the correlation coefficient began to fall, the first value at which the residual sum of squares (RSS) curve presented an inflection point, and through the direct visual inspection of the consistency matrix, the best *r* value was selected. Finally, *r* = 2 met all the predefined quality criteria; in other words, gastrointestinal cancer could be divided into two subgroups (Figure 1(a)). Of the 1,043 gastrointestinal samples from the TCGA, 291 were classified into the hypoxia group and 752 were classified into the normoxia group. The hypoxia group consisted of an approximately equal number of CRC and STAD samples, and there was a moderately higher number of CRC samples than STAD samples in the normoxia group (Figure 1(b)), indicating that the hypoxia-driven pattern was similar in tumors of the two different tissue types. In addition, to further consolidate the hypoxia-oriented subtyping of gastrointestinal cancer, PCA was adopted to inspect the subtypes identified by the NMF algorithm. The results showed a highly consistent trend; that is, hypoxic CRC and STAD had relatively higher similarities than their respective normoxic counterparts (Figure 1(c)).

To validate the two hypoxia-driven subtypes identified in the TCGA cohort, two independent cohorts, GSE39582 (CRC, 585 samples from the French Ligue Nationale Contre le Cancer) and GSE62254 (STAD, 300 samples from the Asian Cancer Research Group), were used to explore the clustering method. Notably, two hypoxia-oriented clusters were also observed in these validation datasets, with a ratio of approximately 1 : 3, and this trend was true for both CRC and STAD (Figure 1(d)–1(f)).

### 3.2. Hypoxia Was Correlated with Poor Survival

Researchers have proposed that hypoxia always leads to an obviously shorter overall survival (OS) interval in cancer patients [16]. Here, we investigated this correlation in gastrointestinal cancer patients. The results showed that hypoxia was significantly correlated with worse OS in both the TCGA (log-rank *P* = 0.3.41e−07, Figure S1(a)) and the GEO dataset (log-rank *P* = 1.8e−06, Figure S1(b)). The 5-year OS rates were 28% (95% CI, 19% to 42%) in the hypoxic group vs. 58% (95% CI, 52% to 66%) in the normoxic group in the TCGA dataset and 51% (95% CI, 45% to 59%) vs. 67% (95% CI, 63% to 71%) in the GEO cohort. In addition, a nearly twofold-higher risk was observed in the hypoxic group than in the normoxic group in both the TCGA (hazard ratio (HR) = 0.52, 95% CI = 0.4–0.67, Cox *P* = 5.2e−07) and GEO (HR = 0.58, 95% CI = 0.46–0.73, *P* = 2.4e−06) cohorts.

### 3.3. Hypoxia Conferred a Higher Level of Immune and Stromal Cell Infiltration but Lower TMB in Tumors

**Figure 2:**
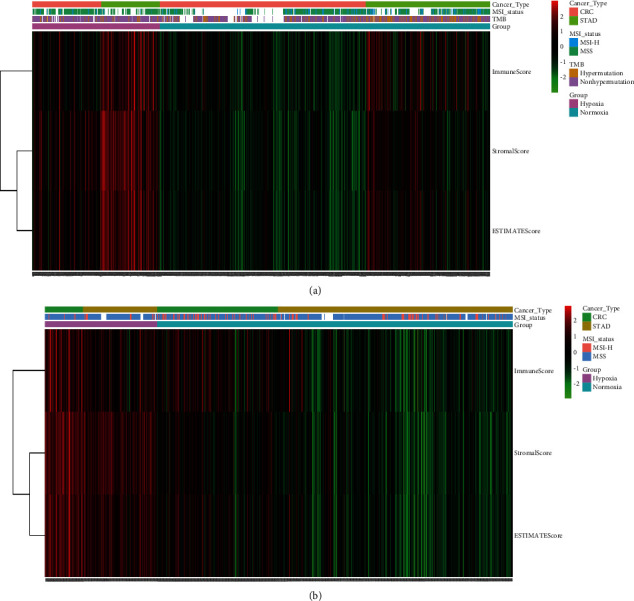
The association of MSI-H, hypermutation, and immune status between the hypoxic and normoxic groups. (a) Immune score, stromal score, and estimate score calculated by the estimate algorithm were visualized by heatmap according to hypoxic and normoxic groups in TCGA cohorts. (b) Immune score, stromal score, and estimate score were visualized by heatmap according to hypoxic and normoxic groups in GEO cohorts. The hypoxic group had a lower TMB but significantly higher immune and stromal cell infiltration.

**Figure 3:**
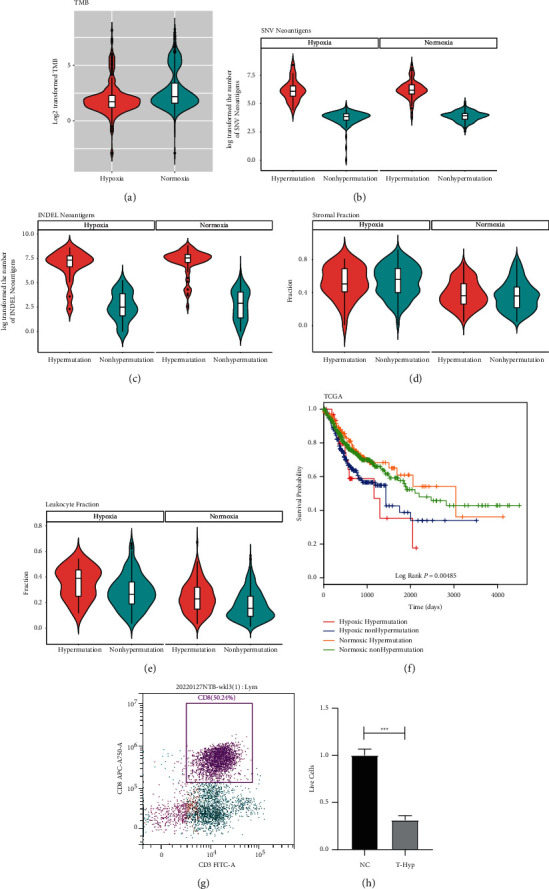
Hypoxic gastrointestinal tumors with low TMB may also have high immune infiltration. (a) Relatively lower TMB was found in the hypoxic group than in the normoxic group. (b) The number of SNV-derived neoantigens was much larger in the hypermutated tumors, irrespective of hypoxic status. (c) The number of INDEL-derived neoantigens was much larger in the hypermutated tumors, irrespective of hypoxic status. (d) The infiltration of stromal cells were much more abundant in the hypoxic group than that in the normoxic group, irrespective of mutation status. (e) The infiltration of immune cells were subjected to regulation by both mutation and oxygen content. (f) Survival differences stratified by the hypoxic status and TMB revealed that the hypoxic-hypermutated group had no survival difference compared to the hypoxic-nonhypermutated group but displayed a worse prognosis than the normoxic-hypermutated and normoxic-nonhypermutated groups in gastrointestinal cancer. (g) Flow cytometry was used to monitor the efficiency of isolation of CD8 T cells. (h) CD8 T cells had higher cytotoxicity than tumor cells under normoxic conditions *in vitro* as evaluated by the T cell cytotoxicity assay.

To decipher the underlying factors associated with worse OS in the hypoxic group, we first compared the immune and stromal cell infiltration levels between the hypoxic and normoxic groups using ESTIMATE. Interestingly, we found that the hypoxic group (both CRC and STAD) had much higher immune and stromal scores than the normoxic group (Figure 2(a) and 2(b)). Further deconvolution of cellular components within tumor tissue revealed a much higher infiltration of macrophages and cancer-associated fibroblasts in the hypoxic group; macrophages and fibroblasts are the two most abundant immune cells and stromal cells, respectively, within tumors (Figure S2). It is important to note that some immune cells, such as Th1 cells and plasma cells, were less infiltrated in spite of a global higher tumoral immunoscores under hypoxic conditions (Figure S2). Since a high TMB has been shown to be closely associated with higher immunogenicity [2], we first compared the TMB between the hypoxic and normoxic groups. Intriguingly, we found that the hypoxic group had a significantly lower TMB than the normoxic group (median mutational frequency 3.3/million bases vs. 4.6/million bases, *P* = 1.483e-11, Wilcoxon test, Figure 3(a)). In addition, a twofold-higher prevalence of high TMB (>10 nonsilent mutations per Mb; 26% VS. 13%) and MSI-H (20% VS. 9%) was observed in the normoxic group compared with the hypoxic group (Figure 3(a)), which explained the lower TMB in the hypoxic group. This obvious contradiction was verified in the GEO datasets (20% vs. 9%, MSI-H), suggesting that external driver forces, such as hypoxia, have a larger effect than intratumoral genetics on TME reshuffling (Figure 3(b)).

To better elucidate the association between immune differences and the TMB in the two subtypes, we further classified the subtypes into hypoxic-hypermutated (35 samples, ∼4%), hypoxic-nonhypermutated (159 samples, ∼18%), normoxic-hypermutated (234 samples, ∼26%), and normoxic-nonhypermutated (461 samples, ∼52%) groups. As expected, the number of predicted neoantigens was closely related to single nucleotide variant (SNV) and insertion-deletion (INDEL)-derived mutations (Figures 3(b) and 3(c)). Of note, stromal infiltration was mainly driven by low oxygen concentrations, instead of by tumor mutations (Figure 3(d)). In contrast, leukocyte infiltration was regulated by both low oxygen concentrations and high TMB; the hypoxic-hypermutated group had increased leukocyte infiltration compared with that of the hypoxic-nonhypermutated group, and both hypoxic groups had increased leukocyte infiltration compared with that of the normoxic-hypermutated and normoxic-nonhypermutated groups, of which infiltration was lowest in the latter group (Figure 3(e)). In other words, the low oxygen concentration had more influence than the TMB because of the higher infiltration level of leukocytes in the hypoxic group. This trend was also observed in patient outcomes. The hypoxic-hypermutated group, although with the highest leukocyte infiltration, had a worse prognosis than the normoxic-hypermutated and normoxic-nonhypermutated groups (Figure 3(e) and 3(f)). Additionally, no significant survival difference was observed between the hypoxic-hypermutated group and the hypoxic-nonhypermutated group (Figure 3(f)).

### 3.4. Enrichment of the Terminally exhCD8 T-cell under Hypoxic Conditions

Given the higher immune score under hypoxic conditions mentioned earlier, we sought to explore the effector T cell status that is closely associated with ICB therapeutic responses. Intriguingly, clinically available ICB targets such as TIGIT, HAVCR2, and CTLA4 showed enhanced expression under hypoxic conditions (Figure S3(a)). In addition, B lymphocyte-induced maturation protein-1 (BLIMP1) and CXCR5, two markers characterized in terminally exhCD8 T cells, both had higher expression levels under hypoxic conditions. These trends were similar in the GEO cohorts (Figure S3(b)). We thus further conceived a custom terminally exhCD8 signature and found that terminally exhCD8 was muchhighly differentiated under hypoxic conditions (Figure S3).

We also checked for the cytotoxic index of effector CD8 T-cell under hypoxic conditions in comparison to that in normoxic conditions and found that a three-fold higher killing effect was observed under normoxic conditions (Figure 3(g) and 3(h)).

### 3.5. Establishment of a Hypoxia-Oriented Gene Expression Model that Separated Gastrointestinal Cancer into High-Risk and Low-Risk Subtypes

**Figure 4:**
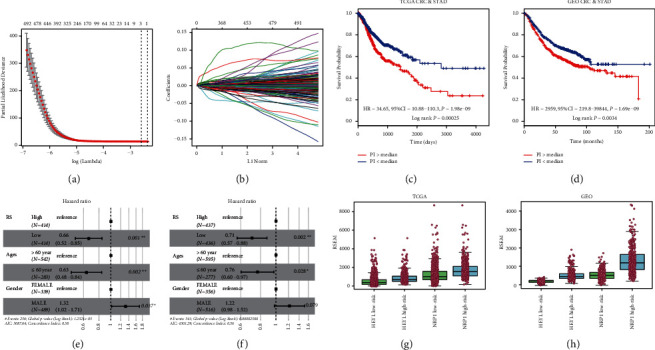
A well-conceivedtwo-gene signature was identified that could predict the prognosis of gastrointestinal cancer patients. (a) Cross-validation for tuning parameter selection in the proportional hazards model. The left vertical dotted line displayed where the CV-error curve hit its minimum (lambda.min) and the right vertical dotted line showed us the most regularized model with CV-error within 1 standard deviation of the minimum (lambda.1se). (b) LASSO coefficient profiles of 1,021 prognosis-associated genes. (c) KM plot of gastrointestinal cancer patients with either high- or low-risk from TCGA after LASSO regression. (d) Validation of the risk model established by TCGA samples in GSE39582 and GSE62254 cohorts. (e) A multivariate Cox PH analysis including risk score, age, and sex of CRC and STAD patients from TCGA. (f) A multivariate Cox PH analysis including risk score, age, and sex of CRC and STAD patients from GEO. (g) The expression of *HEYL* and *NRP1* in the high- or low-risk group is cut off by the median risk score in TCGA. (h) The expression of *HEYL* and *NRP1* in the high- or low-risk group in GEO.

We next sought to identify genes that may serve as prognostic markers in hypoxic and normoxic tumors. First, we identified 1,828 DEGs that were induced by hypoxic conditions. The Cox proportional hazard survival analysis showed that 1,021 DEGs were correlated with the survival outcomes of gastrointestinal cancer patients. Several genes with increased expression were correlated with worse survival outcomes in patients with hypoxic tumors, including *HEYL* (Hes Related Family BHLH Transcription Factor With YRPW Motif Like) (hazard ratio (HR) = 1.421, 95% confidence interval (CI) 1.108–1.824, *P*=0.00571), *PRICKLE1* (Prickle Planar Cell Polarity Protein 1) (HR = 1.391, 95% CI 1.084–1.786, *P*=0.0001094) and *NRP1* (Neuropilin-1) (HR = 1.888, 95% CI 1.463–2.436, *P*=1.01*e* − 06). Additionally, we wanted to determine an expression signature that could separate gastrointestinal patients into two groups with either a high or low prognostic index (PI, see methods for more detail). Notably, gastrointestinal patients could be separated into two groups with either high or low PI based on 1–27 survival-related genes. A well-conceivedtwo-gene signature (*HEYL*^*∗*^9.71E−05 and *NRP1*^*∗*^2.07E−05) was identified that could predict the prognosis of gastrointestinal cancer patients in the TCGA cohort (HR = 34.65, 95% CI = 10.88–110.3, *P*=1.98*e* − 09, log-rank*P*=0.00025; Figure 4(a)−4(c)), and the model was validated in another independent GEO dataset (HR = 2959, 95% CI = 219.8–39844, *P*=1.98*e* − 09, log rank *P*=0.0034; Figure 4(d)), suggesting the robustness of this two-gene signature. A multivariate Cox PH analysis including risk score, age, and sex of CRC and STAD patients was further carried out, and the 2-gene prognostic signature remained significant after controlling for age and sex (Figure 4(e) and 4(f)). The 2-gene prognostic signature, coupled with TMB, also revealed that the high-risk hypermutated group had a poorer outcome than the low-risk hypermutated and low-risk nonhypermutated groups (FigureS4), which was in line with the hypoxia plus TMB comparison (Figure 3(f)). The expression levels of both *HEYL* and *NRP1* were significantly higher in the high-risk group than in the low-risk group (Figure 4(g) and 4(h)). In addition, all ICB targets showed enhanced expression in the high-risk group (Figure S5), which was consistent with the hypoxia-normoxia comparison (Figures S3(a) and S(b)).

## 4. Discussion

In this study, we tried to decipher the relationship between immune score and TMB under hypoxic conditions and found that the hypoxic group had a significantly lower TMB but much higher immune and stromal scores (Figure 3), suggesting that a high TMB is not always related to high immunogenicity, and a high immune score alone does not always correlate with a favorable outcome. It is tempting to believe that the abundant tumor-supporting stromal cells neutralize the advantage imparted by antitumor immune cells under hypoxic conditions, but this warrants further investigation. Additionally, it must be pointed out that some immune cells that boost antitumor responses such as Th1 and plasma cells, were less infiltrated under hypoxic conditions (Figure S2), although qualifying for a higher total immune score. In contrast, terminally exhCD8 T cells were enriched under hypoxic conditions. As such, the current immunoscoring system may not suit all tumors and needs optimization (e.g., plus hypoxic score weightiness) to improve its performance in prognosis prediction accuracy and select tumor patients who would benefit from immunotherapy [17]. It should be noted that though helicobacter pylori and Epstein–Barr virus infection are closely associated with gastric cancer [18], no significant differences were observed between the hypoxic and normoxic groups (data not shown). Bhandari et al. [19] found that elevated hypoxia was associated with increased mutational load across cancer types, which was different from the observation in this study. Further, an inspection found that the reason may be ascribed to sampling differences because only 51 CRC and 29 STAD samples were enrolled in their study [19].

Hypoxia has been shown to be an inhibitory factor for immunotherapy, but the intrinsic mechanism is poorly understood. Increased HIF‐1 levels in hypoxic regions lead to the inhibition of T-cell activity via the upregulation of PD-L1 and result in immune suppression [20]. In this study, in addition to PD-L1, a number of ICB targets showed elevated expression under hypoxic conditions (Figure S3), which was consistent with the hypothesis that hypoxia favors glycolytic anaerobic metabolism by the HIF1*α*-dependent promotion of T-cell receptor (TCR) signaling, including but not limited to increased immune checkpoint molecule levels (both activators and inhibitors) [21, 22]. Under hypoxic conditions, the cytotoxic capacity of CD8 T cells was weakened, reconciling the seemingly opposite evidence regarding the higher infiltration of immune cells and poor outcomes [21, 23]. Thus, targeting hypoxia (e.g., TH-302) is a promising therapeutic option for reversing immunosuppressive TME [24]. Recently, more and more evidence has shown vegetables such as Allium sativum or cepa in particular, as well as their constituents and extracts, as a potential therapeutic strategy in gastric cancer and colon cancer due to their effects on immune function modulation by activating T-cell proliferation [25]. In this manner, the synergistic combination of ICB, dietary therapy, and hypoxia-oriented inhibitor treatment may substantially improve the immune status of gastrointestinal cancer patients and prolong their survival.

Finally, we conceived and validated a two-gene hypoxic signature (*HEYL* and *NRP1*) predicting the prognosis of gastrointestinal cancer patients. High expression of HEYL has been shown to accelerate gastric carcinoma development [26]. NRP1 is a marker for CD4+ regulatory T cells, is sometimes coexpressed with PD-1 on a subset of CD8 tumor-infiltrating T lymphocytes, and inhibits T cell antitumor immunity [27]. In this study, elevated NRP1 and immune checkpoints such as PD1 were observed under hypoxic conditions and in the two-gene-basedhigh-risk group (Figure 4(g) and 4(h)), (Figure S5), which suggests a close association between NRP1 and the hypoxia-derived immunosuppressive TME. As a novel immune memory checkpoint, blockade of NRP1 may enhance long-livedtumor-specific memory T cells, which are important for durable antitumor immunity [28]. The two-gene hypoxic signature, together with immune status detection, provides a personalized therapeutic schedule to improve the curability of gastrointestinal cancer.

## 5. Conclusions

A high immune score driven by hypoxia was not associated with a favorable outcome in gastrointestinal cancer. A more subtle immune score system should be refined, considering external factors such as the hypoxia index. Thus, the combination of ICB and hypoxia-oriented inhibitors may greatly improve the therapeutic effect and prolong the survival of gastrointestinal cancer patients.

## Data Availability

All datasets presented in this study are included in the article.

## Supplementary Materials

Supplementary Materials Supplementary figure 1. Survival differences between the hypoxic and normoxic groups in gastrointestinal cancer. A. KM plot of OS status for samples from TCGA. B. KM plot of OS status for samples from GEO. Supplementary figure 2. The relative infiltration levels of 64 immune and stromal cells within gastrointestinal tumor tissue between the hypoxic and normoxic groups of TCGA (A) and GEO (B), respectively. Supplementary figure 3. The expression levels of ICB targets and exhausting scores between the hypoxic and normoxic groups. A. Violin plots showing canonical ICB targets and exhausting scores stratified by hypoxic conditions in TCGA samples. B. Violin plots showing canonical ICB targets and exhausting scores stratified by hypoxic conditions in GEO samples. The median is depicted as a horizontal line splitting the main box in half. Supplementary figure 4. Survival differences stratified by the 2-gene prognostic signature and TMB revealed that the high-risk hypermutated group had a worse prognosis than the low-risk hypermutated and low-risk nonhypermutated groups in gastrointestinal cancer from TCGA. Supplementary figure 5. The expression levels of ICB targets between the high- and low-risk groups according to risk scores derived from the two-gene prognostic signature. A. Violin plots show canonical ICB targets stratified by risk score in TCGA samples. B. Violin plots show canonical ICB targets stratified by risk score in GEO samples. The median is depicted as a horizontal line splitting the main box in half.
