# Supplementary material for: Hypoxia Confers Tumor with a Higher Immune Infiltration but Lower Mutation Burden in Gastrointestinal Cancer
Source: J Oncol. 2022 Sep 12;2022:4965167. doi: 10.1155/2022/4965167 (PMC9484921; doi:10.1155/2022/4965167)
Supplement: Supplementary Materials — Supplementary figure 1. Survival differences between the hypoxic and normoxic groups in gastrointestinal cancer. A. KM plot of OS status for samples from TCGA. B. KM plot of OS status for samples from GEO. Supplementary figure 2. The relative infiltration levels of 64 immune and stromal cells within gastrointestinal tumor tissue between the hypoxic and normoxic groups of TCGA (A) and GEO (B), respectively. Supplementary figure 3. The expression levels of ICB targets and exhausting scores between the hypoxic and normoxic groups. A. Violin plots showing canonical ICB targets and exhausting scores stratified by hypoxic conditions in TCGA samples. B. Violin plots showing canonical ICB targets and exhausting scores stratified by hypoxic conditions in GEO samples. The median is depicted as a horizontal line splitting the main box in half. Supplementary figure 4. Survival differences stratified by the 2-gene prognostic signature and TMB revealed that the high-risk hypermutated group had a worse prognosis than the low-risk hypermutated and low-risk nonhypermutated groups in gastrointestinal cancer from TCGA. Supplementary figure 5. The expression levels of ICB targets between the high- and low-risk groups according to risk scores derived from the two-gene prognostic signature. A. Violin plots show canonical ICB targets stratified by risk score in TCGA samples. B. Violin plots show canonical ICB targets stratified by risk score in GEO samples. The median is depicted as a horizontal line splitting the main box in half. [file 4965167.f1.pdf]

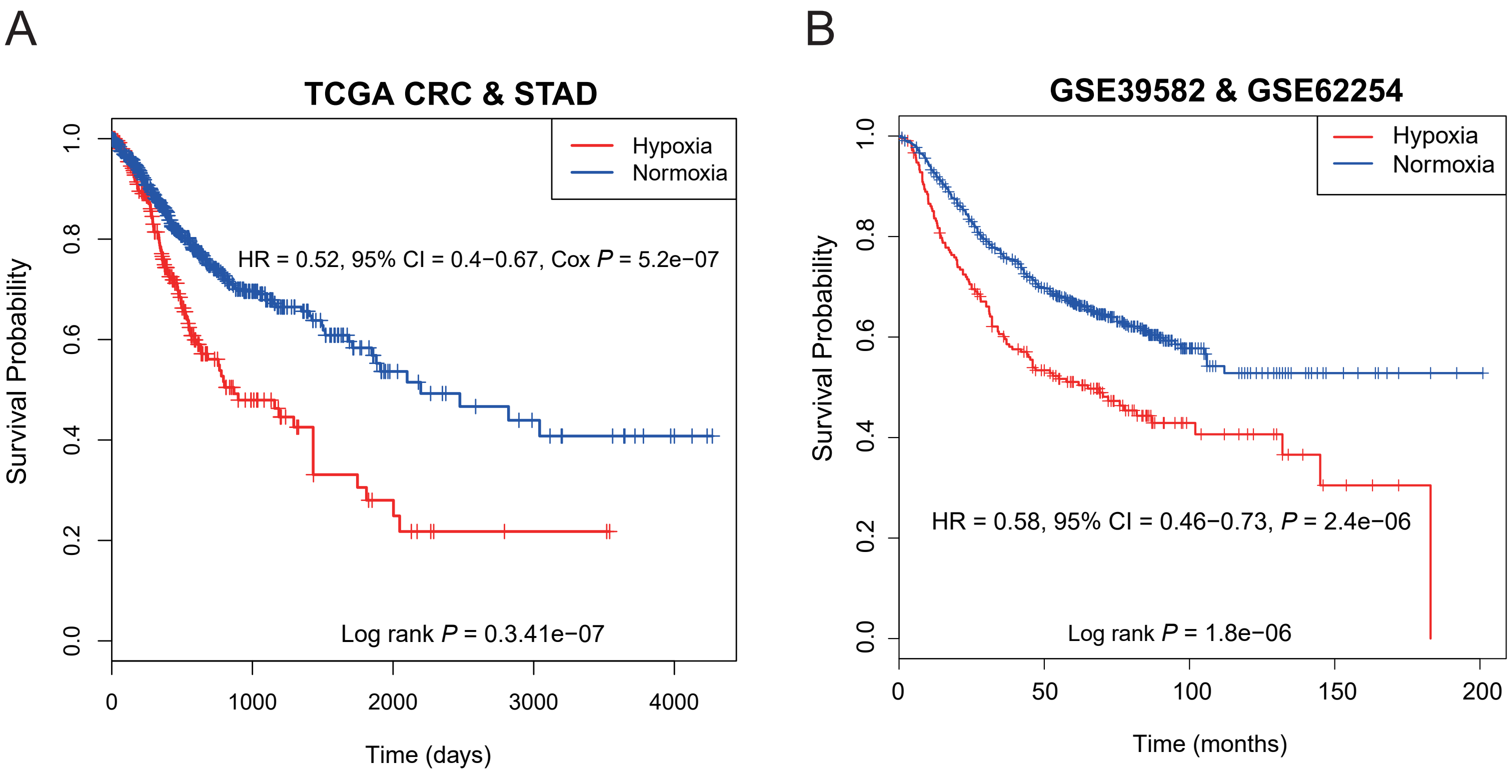

Supplementary figure 1. Survival differences between the hypoxic and normoxic groups in gastrointestinal cancer. A. KM plot of OS status for samples from TCGA. B. KM plot of OS status for samples from GEO.

A

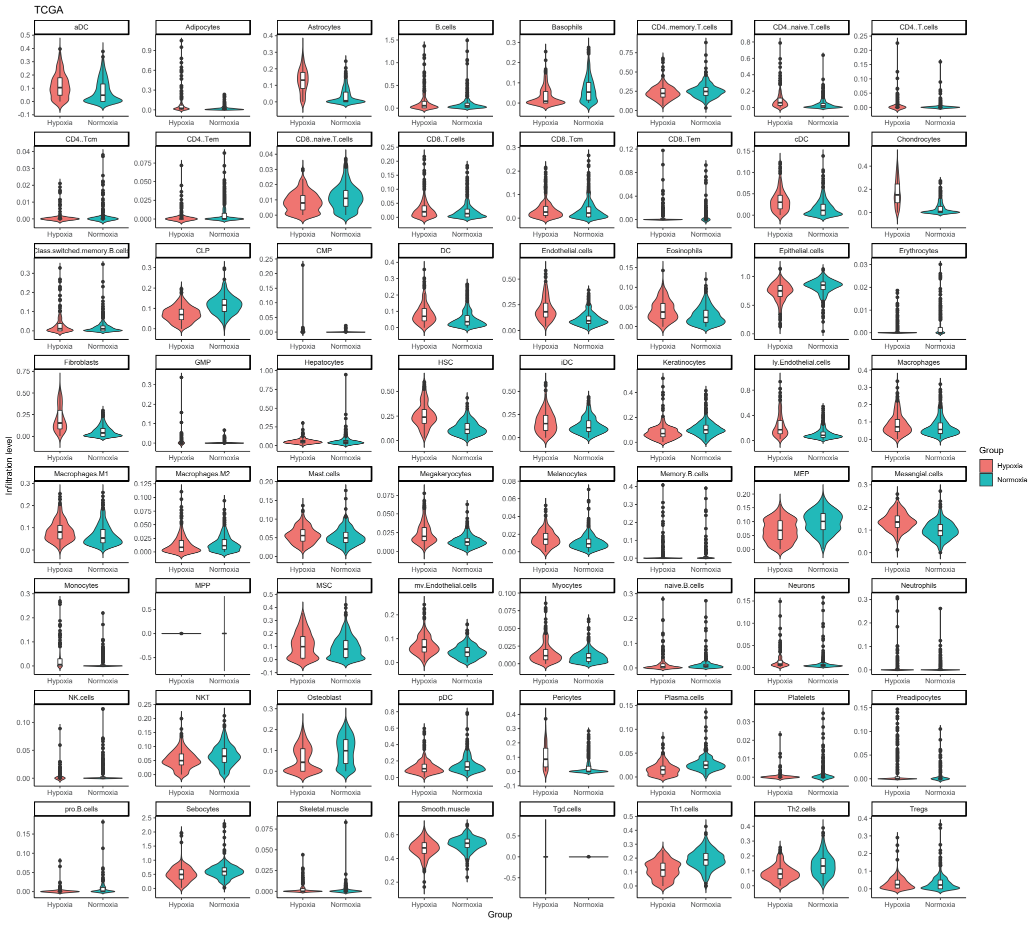

B

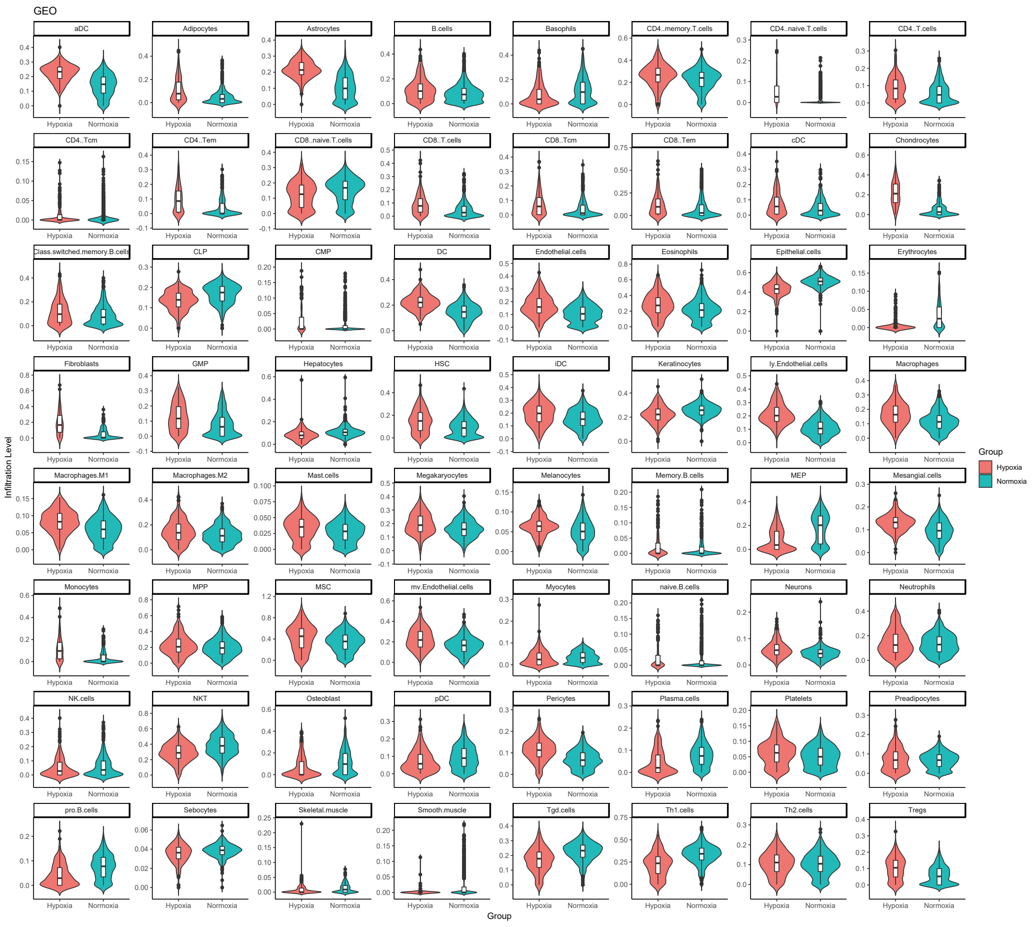

Supplementary figure 2. The relative infiltration levels of 64 immune and stromal cells within gastrointestinal tumor tissue between the hypoxic and normoxic groups from TCGA (A) and GEO (B), respectively.

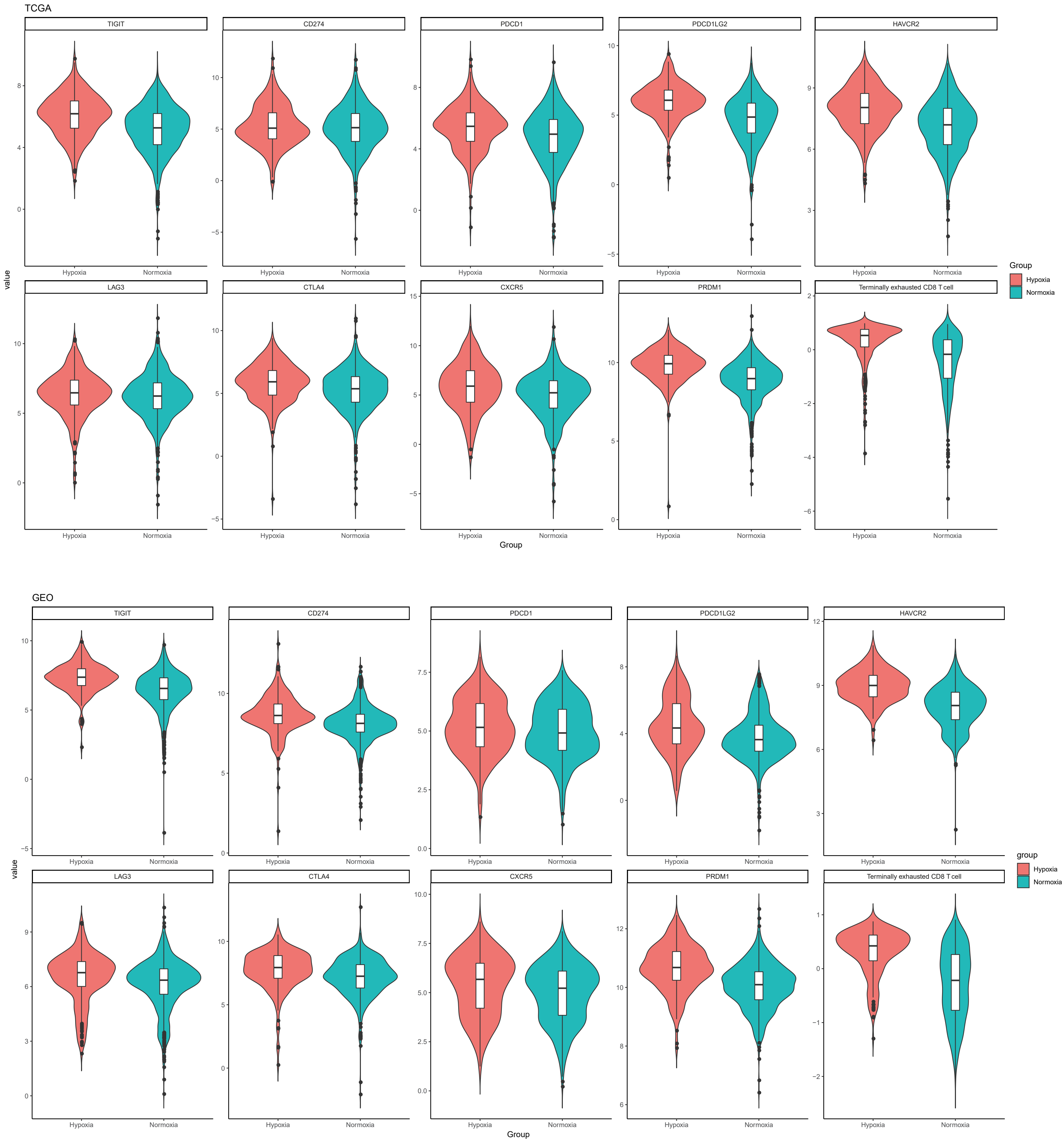

Supplementary figure 3. The expression levels of ICB targets and exhausted scores between the hypoxic and normoxic groups. A. Violin plots showing canonical ICB targets and exhausted scores stratified by hypoxic conditions in TCGA samples. B. Violin plots showing canonical ICB targets and exhausted scores stratified by hypoxic conditions in GEO samples. The median is depicted as a horizontal line splitting the main box in half.

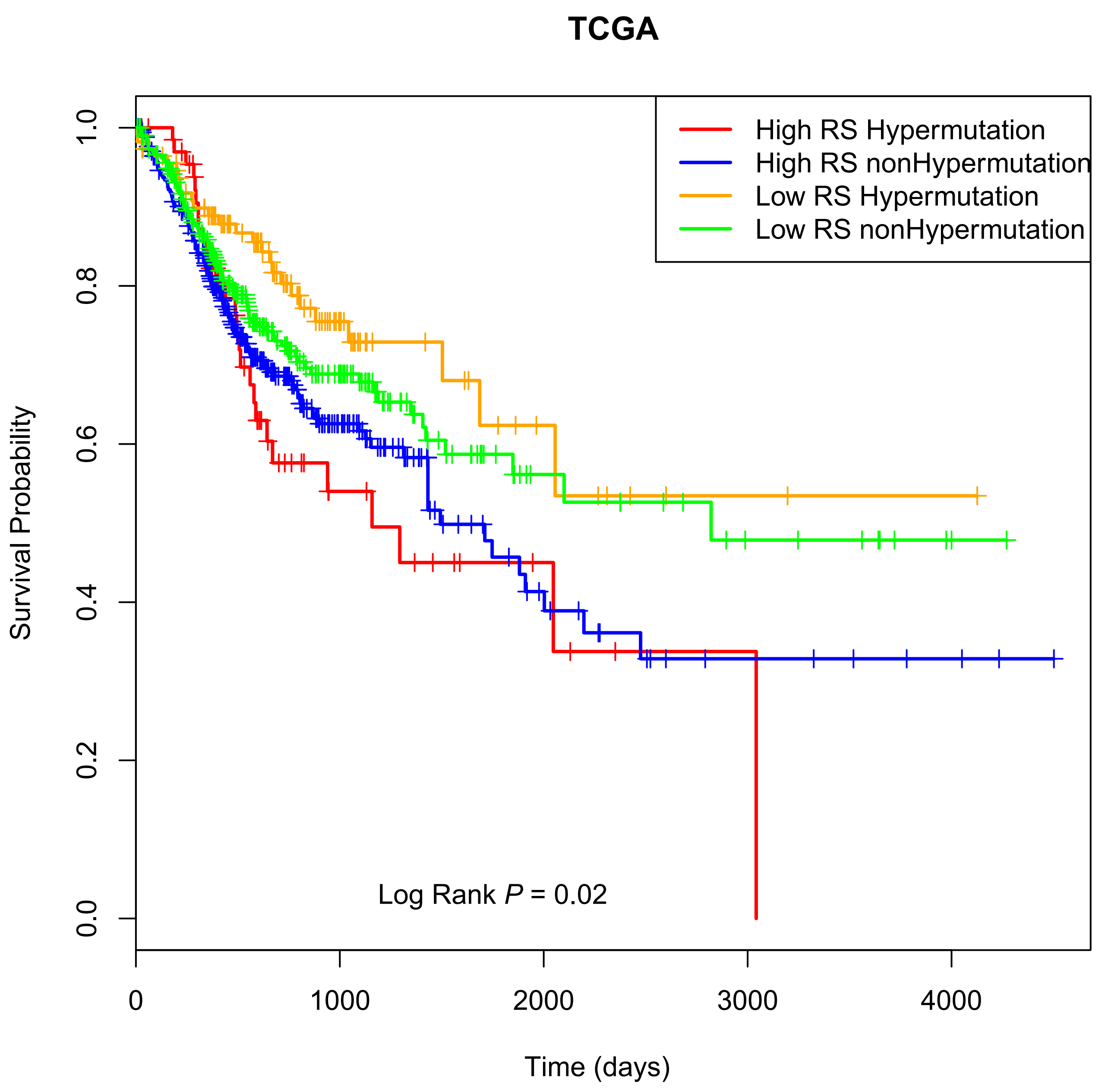

Supplementary figure 4. Survival differences stratified by the 2-gene prognostic signature and TMB revealed that the high-risk hypermutated group had a worse prognosis than the low-risk hypermutated and low-risk nonhypermutated groups in gastrointestinal cancer from TCGA.

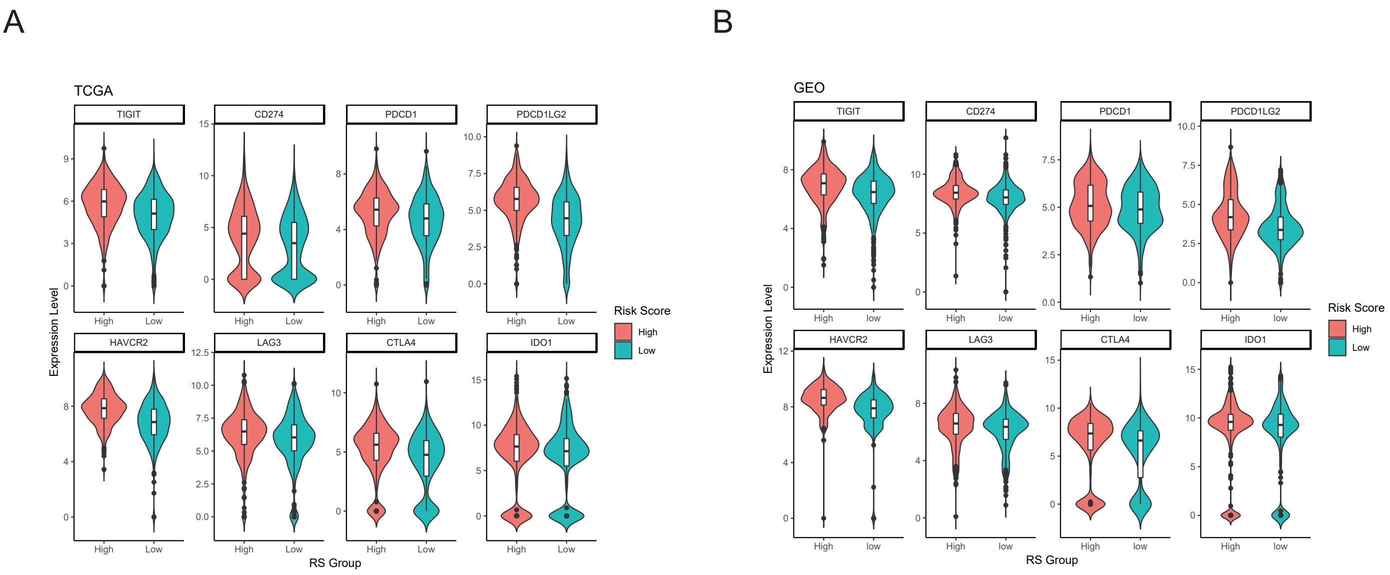

Supplementary figure 5. The expression levels of ICB targets between the high- and low-risk groups according to risk scores derived from the two-gene prognostic signature. A. Violin plots showing canonical ICB targets stratified by risk score in TCGA samples. B. Violin plots showing canonical ICB targets stratified by risk score in GEO samples. The median is depicted as a horizontal line splitting the main box in half.
